# The Interplay Between Theory of Mind and Social Emotional Functioning in Adolescents With Communication and Language Problems

**DOI:** 10.3389/fpsyg.2019.01488

**Published:** 2019-07-03

**Authors:** Lidy Smit, Harry Knoors, Daan Hermans, Ludo Verhoeven, Constance Vissers

**Affiliations:** ^1^Royal Dutch Kentalis, Kentalis Academy, St. Michielsgestel, Netherlands; ^2^Behavioural Science Institute, Learning and Plasticity, Radboud University, Nijmegen, Netherlands

**Keywords:** developmental language disorder, deaf and hard of hearing, theory of mind, adolescents, social emotional functioning

## Abstract

Adolescents with developmental language disorders (DLDs) and adolescents who are deaf or hard of hearing (D/HH) are at greater risk of social emotional problems. These problems may not only be attributed to communication and language problems but, at least in part, to Theory of Mind (ToM) deficits as well. In this mini review, an overview is provided of studies on social emotional functioning and ToM performance in adolescents with DLD and D/HH adolescents. A possible interplay between social emotional functioning and Theory of Mind is discussed. There is empirical evidence for social emotional problems and ToM problems in both adolescents with DLD and D/HH. We hypothesized that language deficits as seen in adolescents with DLD and impoverished exposure to language and communication, as seen in adolescents who are D/HH can explain differences in social emotional functioning and ToM performance. The present mini review provides a possible framework for the relation between ToM and social emotional functioning in adolescents with communication and language problems, which is mediated by their limited linguistic ability or restricted language exposure and gives suggestions for future research.

## Introduction

From an empirical perspective, we know that children with DLD and D/HH suffer from social difficulties and emotional problems much more often than their typically developing (TD) peers (Stanzione and Schick, 2014; Vissers and Koolen, 2016; Vissers and Hermans, 2018).

Within a neuropsychological framework, the causes underlying the manifestation of social emotional problems in adolescents with DLD and D/HH are thought to be rooted in cognitive deficits such as Theory of Mind or Executive Functioning (e.g., Morgan and Lilienfeld, 2000; Vissers and Hermans, 2018).

The ability to represent mental states of oneself and others in order to understand and predict behavior is defined as Theory of Mind (ToM). ToM was first introduced in the 1970s in primate research by Premack and Woodruff (1978). The term “theory” is used for two reasons. First you cannot directly see what one knows, believes, or thinks and hence you cannot directly observe mental states. What you can do is make an assumption and a deduction from the context (e.g., nonverbal and verbal behavior) and form a theory of the mental states. The second reason the term theory is used is that you can use mental state information to predict the behavior of the other. The term mind refers to mental states, deduced from facial expression, physical movements, and linguistic expressions. Thus, theory of mind is an abstract representation of mental states and we make predictions based on these mental representations. With a well-developed ToM, we can understand the goals of others and predict their (Perner, 1991). Therefore, ToM is indispensable for the adequate adjustment of our own behavior in social situations.

We consider socio-emotional problems as observable behavior and ToM as the cognitive process that underlies this behavior, fitting into the neuropsychological ideology (e.g., Berlucchi, 2010).

Almost all research has focused on the ToM functioning of children in elementary school age and hardly any on adolescents (Hughes and Devine, 2015). In our opinion a serious omission, because a primary challenge in adolescence involves the formation of new social relationships. Many social experiences occur in a completely novel context (Ballonoff Suleiman and Paige Harden, 2016). In addition to the pleasure of doing things together and communicating with others who experience the same things, a first function of social behavior is to practice roles that prepare for adulthood. Secondly, peers function as a shelter and offer support and solidarity (Siegel and Shaughnessy, 1995).

However, the social emotional domain is not the only domain that changes in the transition to adolescence. From a neural perspective, there is evidence that brain areas become more specialized throughout middle childhood into adolescence, especially the areas that are used for reasoning about mental states (Lagattuta et al., 2015). In the domain of cognition, there is evidence that ToM gradually develops during childhood and continues to develop in later childhood (Choudhury et al., 2006; Peterson and Wellman, 2018).

The association between ToM competence and social emotional functioning is supported within normative samples of young children (Razza and Blair, 2009; Shakoor et al., 2011; Slaughter et al., 2015; Białecka-Pikul et al., 2017). For example, better ToM outcomes in preschool predict better social competence in kindergarten (Razza and Blair, 2009). In a similar vein, poor ToM during childhood predicted becoming a victim of bullying or a bully in early adolescence (Shakoor et al., 2011).

Studies of ToM performance and the interplay with social emotional functioning are especially relevant for two groups of adolescents, those with DLD or those who are D/HH, where there is a high risk of social emotional problems. But there is a paucity in research on ToM performance in these adolescents, so we decided to map the little research that has been done.

In the first part of this mini review, we will focus on studies in the area of ToM performance and social emotional functioning in adolescents with DLD or those who are D/HH. We define adolescence as the transitional phase of growth and development between childhood and adulthood, ranging from 10 to 24 years. However, due to a lack of research within the target groups, we have also included studies of older age groups to provide us with a better insight into the possible developmental course.

## Mini Review

### Social Emotional Functioning and Theory of Mind Performance in Adolescents With Developmental Language Disorder

#### Social Emotional Functioning

Studies in the field of social emotional functioning in adolescents with DLD have focused on a number of areas. Some of these studies have focused on symptoms as anxiety and depression. Adolescents with DLD (19–25 years) are reported to experience more social anxiety and depressive symptoms and endorsed higher levels of social interaction anxiety symptoms at age 31 (Brownlie et al., 2015).

Anxiety symptoms are primarily related to the quality of social interaction and communication. Having more peer problems was found to be associated with more depressive symptoms at 16 and 17 years in the adolescents with DLD but these associations were not apparent in typically developing adolescents. Reading skills and expressive language were related only to behavioral problems such as hyperactivity, not to emotional problems. Pragmatic abilities were related to behavioral, emotional, and social difficulties. It is concluded that having DLD puts an individual at greater emotional risk (Wadman et al., 2011). In a systematic review and meta-analysis on the incidence and severity of emotional problems in later adolescence, it was evidenced that DLD adolescents were about two times more likely to show disorder levels of overall internalizing problems, overall externalizing and ADHD symptoms than TD peers (Yew and O’Kearney, 2013). Adolescents with DLD suffer from emotional problems like low self-esteem, symptoms of anxiety and depression, hyperactivity, conduct problems, and bullying and they thereby report a higher level of peer problems (Wadman et al., 2008, 2011; Conti-Ramsden et al., 2013). There is evidence from a longitudinal study that young adolescents with DLD enter adulthood with lower rates on self-questionnaires on social self-efficacy and self-esteem and higher rates on shyness, in comparison to their typically developing peers (TD) (Durkin et al., 2017).

A few studies exist that focus on problems that arise in peer relationships. A study in which 171 children with DLD were followed from the age of 7 to 16 shows that most of them experience problems in social contacts with peers, which was related to pragmatic language problems. Pragmatic skills are vital for communicating our personal thoughts, ideas, and feelings. These social emotional problems seem to persist until later age. Importantly, if the problems start to occur for the first time at the age of 14, the problems quickly appear to increase rapidly in proportion (Mok et al., 2014). Adolescents with DLD are more likely to exhibit poorer quality of their friendships in comparison to their typically developing (TD) peers. A mix of expressive and receptive problems in some adolescents with DLD is indicative of a more severe impairment and seems to be predictive of friendship quality in adolescence (Durkin and Conti-Ramsden, 2007). In a longitudinal study by St Clair et al. (2011), it was outlined that from the age of 7 to 16, there is an increase in social (peer) problems by adolescents with DLD, possibly due to the increasing central role of language in peer relations during adolescence. By 16 years of age, nearly 40% of individuals with a history of DLD appear impaired in their interactions with peers (St Clair et al., 2011). Adults in the age of 34 with DLD report problems in the area of expressing themselves and problems in receiving information in conversations. Participants report that these problems affect their subjective well-being the most (Arkkila et al., 2008).

#### Theory of Mind

Although ToM can be a predictor for social functioning and many adolescents with DLD experience social emotional problems, studies dealing with ToM functioning in adolescents with DLD are scarce. Cognitive ToM tasks such as the false belief task or the storytelling task are mostly used in research. These tasks are highly verbal and could be very difficult for adolescents with DLD. A cohort study in which 17 men with a severe receptive DLD in childhood (4–9 years) were reassessed in middle childhood (early 20s) and early adult life and again in their mid-30s revealed that these men scored lower than TD peers and siblings on three ToM tasks, namely strange stories task, awkward moments task, and reading the eyes in the mind task (Clegg et al., 2005).

### Social Emotional Functioning and Theory of Mind Performance in Deaf or Hard of Hearing Adolescents

#### Social Emotional Functioning

Most of what we know so far about social emotional functioning in people who are D/HH comes from studies done in children. Little research has been done on social emotional functioning in D/HH adolescents. Empirical evidence from the few studies available indicates that, overall, D/HH students reported increased levels of mental health problems compared with hearing peers, ranging from 32,6 to 54 percent. Specific to depression, deaf adolescents appear to be dealing with higher rates compared to their TD peers (Margaret Brown and Cornes, 2015). Other examples of mental health problems given in this study are internalizing problem behavior, externalizing problem behavior, and anxiety. Reasons given by the researchers for these problems are limited incidental learning and social difficulties such as misinterpreting social information.

Van Gent et al. (2007) examined psychopathology in deaf adolescents (13–21 years) using checklist assessments by parents [Child Behavior Checklist (CBCL)], teachers [Teacher’s Report Form (TRF)], semi-structured clinical interviews with adolescents [Semi-structured Clinical Interview for Children and Adolescents (SCICA)], and expert ratings of dossier data. It was concluded that there was a high prevalence of psychopathology, emotional problems, and behavioral problems. In a study that compared psychosocial risk behaviors of adolescents who are D/HH with those of their hearing peers, significant differences emerged between groups. Adolescents that were D/HH demonstrated clinically higher scores than their hearing peers on psychosocial risk behaviors of risk to others, social and adaptive functioning, need for structure, aggression toward people and animals, destruction of property, theft and deceit, and rules violations (Coll et al., 2009).

Adolescents that are D/HH show higher rates of mental distress according to parental ratings on the Strengths and Difficulties Questionnaire (SDQ) in comparison to their hearing peers, whereas self-reports show elevated scores only in peer relationship problems in comparison to their hearing peers. If sign language and/or oral language abilities are good, the children do not have a substantially higher level of psychosocial difficulties than their hearing peers (Fellinger et al., 2009; Dammeyer, 2010). Adolescents up to 21 years with cochlear implants are likely to show an elevated rate of emotional and behavioral problems in different areas measured with the SDQ, with the most difficulties in the area of peer problems with the risk of isolation (Stevenson et al., 2015). A study that examined social emotional functioning in terms of everyday problems among D/HH adolescents across various life domains found that adolescents perceived the greatest stress related to the future, peers, and school, in that order. Considerably less stress was experienced with regard to parents, leisure, and romantic relationships. Lower pragmatic abilities and an increased level of withdrawal coping style were found to be associated with higher levels of perceived stress (Zaidman-Zait and Dotan, 2017). Hintermair (2013) assessed D/HH students with a mean age of 12.4 years and found that increased problems with executive functions are related to lower levels of communicative competence in students and higher probability of behavioral problems. Better communicative competence is also correlated with improved social emotional development.

In a study of mental disorders in D/HH students, the only significant predictive background variable was the language used at home. The students also reported significant thought problems that can be associated with a lack of self-reflection, inner speech, and emotional self-regulation. The only significant predictive background variable for mental health issues in this study was the language used at home (Margaret Brown and Cornes, 2015).

#### Theory of Mind

Up to now, most research has focused on false belief understanding in D/HH adolescents using false belief tasks as a benchmark. Most empirical evidence so far suggest cognitive ToM delays, based on false belief tasks, in persons that are D/HH and have hearing parents (Marschark, 1993; see Peterson and Siegal, 2000, for a review; Meristo et al., 2007). Russell et al. (1998) found significant improvement on first-order false belief reasoning (using the false belief task) at the age of 13–16 years. The false belief task requires recursive understanding of A’s false belief about B’s belief.

Using the Look Prediction Task and the Strange Stories task and the Reading The Mind in The Eyes Test, Lecciso et al. (2016) found that late signers and oral deaf adults (age range: 15–28 years) have impairments in both social-perceptual and social cognitive components of ToM in comparison to TD peers. Marschark et al. (2018) assessed ToM performance in 94 deaf adolescents attending university by evaluating their understanding of sarcasm and advanced false belief (second-order false belief and double bluff), and consistent with the previous studies, deaf participants scored lower than hearing peers. The participants were young adults with language skills appropriate for university entrance. The authors state in their research that it appears unlikely that the differences in ToM performance observed in this study can be attributed to linguistic ability. In contrast to previous studies that state that early access to sign language predicts better ToM performance, the present findings indicate that neither being a native signer nor having access to spoken language *via* a CI at an earlier age is associated with ToM performance among college students.

## Discussion

In adolescents with DLD, we see the manifestation of social emotional problems. They report higher social anxiety, depressive symptoms, behavioral problems, social and emotional difficulties, internalizing problems, low self-esteem, conduct problems, and peer problems (e.g., Wadman et al., 2011; Conti-Ramsden et al., 2013; Yew and O’Kearney, 2013; Brownlie et al., 2015). What the exact, underlying psychological predictors are that lead to a greater risk for social emotional problems is not mentioned (Wadman et al., 2011; Mok et al., 2014; Brownlie et al., 2015). Studies that link and explore both underlying cognitive psychological mechanisms, such as ToM, and the manifestation of social emotional symptoms on a possible interplay are nonexistent in adolescents with DLD.

We actually see the same trend in studies on DHH adolescents. Studies provide information about the manifestation of social emotional functioning in DHH adolescents, saying that there are more and bigger social emotional problems in comparison to their hearing to their hearing peers—varying from problems in internalizing problem behavior, externalizing problems, anxiety, depression, peer problems, aggression problems toward peers, higher perceived stress levels, risk for social isolation (e.g., Van Gent et al., 2007; Coll et al., 2009; Fellinger et al., 2009; Dammeyer, 2010; Margaret Brown and Cornes, 2015). But the cause of these problems is not sought in underlying psychological mechanisms. There are no studies that investigate an interplay between ToM functioning and social emotional outcomes in adolescents that are DHH.

There are only few studies that focus on ToM functioning in adolescents with DLD or that are DHH. In both the studies done in DLD and DHH adolescents, lower scores on ToM tasks are found in comparison to their hearing peers (Marschark, 1993; see Russell et al., 1998; Peterson and Siegal, 2000; Meristo et al., 2007; Marschark et al., 2018). These studies only look at ToM functioning and do not relate the results to possible behavioral manifestations.

Several important theoretical questions remain open. It is not clear if the social emotional problems in adolescents in DLD and adolescents that are DHH can be explained by ToM deficits. But suppose this turns out to be the case, in what way? Vissers and Koolen (2016) proposed three models to explain the interplay between ToM, language, and social emotional functioning. In the first, ToM facilitates language development. ToM is proposed to allow children to learn new words through their sensitivity to referential intentions of others. To support this model, we have to look back to preschool where the roots of ToM are formed. The few studies on young children with DLD show that they perform more poorly on ToM tasks than their TD peers (Farrant et al., 2006; Andrés-Roqueta et al., 2013, 2016; Jester and Johnson, 2016).

The second model argues that language stimulates ToM development. There is evidence for the conversational approach, proposing that ToM development is positively influenced by conversational interactions about events and facets of the external perceptual world as well as conversations about inner thoughts and states. Deaf children from hearing families often fail on ToM tasks (see Peterson and Wellman, 2009; Spencer, 2010, for reviews). But deaf children of deaf parents using sign language perform the same on ToM tasks as their TD peers (e.g., Peterson and Siegal, 2000; Peterson et al., 2005; Schick et al., 2007).

Studies point out the importance of communication—no matter the modality or degree of hearing loss—for the psychosocial well-being of persons that are D/HH (Fellinger et al., 2009; Dammeyer, 2010). Russell et al. (1998) found significant improvement on first-order false belief reasoning at the age of 13–16 years. Explanation that is given for this ongoing development could be the wider communicative experience within and outside the family, which would support this second model. Higher rates of social emotional problems in adolescents with DLD and adolescents that are D/HH are linked to barriers in communication (Van Gent et al., 2007; Coll et al., 2009; Brownlie et al., 2015).

The third model affirms that language deficits and ToM deficits co-occur because they are fueled by a single neuropsychological underlying structure for instance such as working memory (WM) an aspect of executive functioning. Future research might focus on these models in adolescents with DLD and that are D/HH. With respect to the foregoing models, the social emotional problems, experienced by both adolescents that have DLD and adolescents that are DHH, can possibly be explained by ToM deficits, which is mediated by their limited linguistic ability, as in the case of adolescents with DLD, or restricted language exposure, as in the case of adolescents that are DHH. But this should be thoroughly investigated; there is currently a big gap between the studies on social emotional functioning and ToM functioning in both target groups.

The models above deserve further evaluation and investigation. Future research could focus on intervention programs to tackle the problems as seen in adolescents with DLD and that are D/HH. But it is also important to test theoretical models that examine the relationship between language and ToM.

## Author Contributions

LS focused on and wrote the empirical part of the mini review and integrated the empirical findings. CV, DH, HK, LV, and LS finalized the mini review.

### Conflict of Interest Statement

The authors declare that the research was conducted in the absence of any commercial or financial relationships that could be construed as a potential conflict of interest.

## References

[ref702] Andrés-Roqueta C. Adrian J. E. Clemente R. A. Katsos N. (2013). Which are the best predictors of theory of mind delay in children with specific language impairment? Int. J. Lang. Commun. Disord. 48, 726–737. 10.1111/1460-6984.12045 24165368

[ref703] Andrés-Roqueta C. Adrian J. E. Clemente R. A. Villanueva L. (2016). Social cognition makes an independent contribution to peer relations in children with Specific Language Impairment. Res. Dev. Disabil. 49, 277–290. 10.1016/j.ridd.2015.12.015 26745788

[ref1] Arkkila E. Rasanen P. Roine R. P. Vilkman E. (2008). Specific language impairment in childhood is associated with impaired mental and social well-being in adulthood. Logoped. Phoniatr. Vocol. 33, 179–189. 10.1080/14015430802088289 18608878

[ref3] Berlucchi G. (2010). “Neuropsychology: theoretical basis” in Encyclopedia of neuroscience. 1001–1006. 10.1016/B978-008045046-9.00996-7

[ref4] Białecka-Pikul M. Kołodziejczyk A. Bosacki S. (2017). Advanced theory of mind in adolescence: do age, gender and friendship style play a role? J. Adolesc. 56, 145–156. 10.1016/j.adolescence.2017.02.009, PMID: 28237631

[ref5] Brownlie E. B. Bao L. Beitchman J. (2015). Childhood language disorder and social anxiety in early adulthood. J. Abnorm. Child Psychol. 44, 1061–1070. 10.1007/s10802-015-0097-5 26530522

[ref6] Choudhury S. Blakemore S.-J. Charman T. (2006). Social cognitive development during adolescence. Soc. Cogn. Affect. Neurosci. 1, 165–174. 10.1093/scan/nsl024, PMID: 18985103 PMC2555426

[ref7] Clegg J. Hollis C. Mawhood L. Rutter M. (2005). Developmental language disorders – a follow-up in later adult life. Cognitive, language and psychosocial outcomes. J. Child Psychol. Psychiatry Allied Discip. 46, 128–149. 10.1111/j.1469-7610.2004.00342.x, PMID: 15679523

[ref8] Coll K. M. Cutler M. M. Thobro P. Haas R. Powell S. (2009). An exploratory study of psychosocial risk behaviors of adolescents who are deaf or hard of hearing: comparisons and recommendations. Am. Ann. Deaf 154, 30–35. 10.1353/aad.0.0074 19569302

[ref9] Conti-Ramsden G. Mok P. L. H. Pickles A. Durkin K. (2013). Adolescents with a history of specific language impairment (SLI): strengths and difficulties in social, emotional and behavioral functioning. Res. Dev. Disabil. 34, 4161–4169. 10.1016/j.ridd.2013.08.043, PMID: 24077068 PMC3830176

[ref10] Dammeyer J. (2010). Psychosocial development in a danish population of children with cochlear implants and deaf and hard-of-hearing children. J. Deaf. Stud. Deaf. Educ. 15, 50–58. 10.1093/deafed/enp024 19745003

[ref11] Durkin K. Conti-Ramsden G. (2007). Language, social behavior, and the quality of friendships in adolescents with and without a history of specific language impairment. Child Dev. 78, 1441–1457. 10.1111/j.1467-8624.2007.01076.x, PMID: 17883441

[ref12] Durkin K. Toseeb U. Botting N. Pickles A. Conti-Ramsden G. (2017). Social confidence in early adulthood among young people with and without a history of language impairment. J. Speech Lang. Hear. Res. 60:1635–1647. 10.1044/2017_JSLHR-L-16-0256, PMID: 28586830 PMC5544415

[ref700] Farrant B. M. Fletcher J. Mayberry M. T. (2006). Specific language impairment, theory of mind, and visual perspective taking. Child Dev. 77, 1742–1853. 10.1111/j.1467-8624.2006.00977.x 17107464

[ref13] Fellinger J. Holzinger D. Beitel C. Laucht M. Goldberg D. P. (2009). The impact of language skills on mental health in teenagers with hearing impairments. Acta Psychiatr. Scand. 120, 153–159. 10.1111/j.1600-0447.2009.01350.x, PMID: 19207129

[ref14] Hintermair M. (2013). Executive functions and behavioral problems in deaf and hard-of-hearing students at general and special schools. J. Deaf. Stud. Deaf. Educ. 18, 344–359. 10.1093/deafed/ent003, PMID: 23418367

[ref15] Hughes C. Devine R. T. (2015). “A social perspective on theory of mind” in Handbook of child psychology and developmental science (volume III): Socioemotional processes. 7th Edn. ed. Lamb M. E. (Hoboken, NJ: Wiley), 564–609.

[ref701] Jester M. Johnson C. J. (2016). Differences in theory of mind and pretend play associations in children with and without specific language impairment. Infant Child Dev. 25, 24–42. 10.1002/icd.1912

[ref16] Lagattuta K. H. Kramer H. J. Kennedy K. Hjortsvang K. Goldfarb D. Tashjian S. (2015). Beyond Sally’s missing marble. Adv. Child Dev. Behav. 48, 185–217. 10.1016/bs.acdb.2014.11.005 25735945

[ref17] Lecciso F. Levante A. Baruffaldi F. Petrocchi S. (2016). Theory of mind in deaf adults. Cogent Psychol. 3:1264127. 10.1080/23311908.2016.1264127

[ref18] Margaret Brown P. Cornes A. (2015). Mental health of deaf and hard-of-hearing adolescents: what the students say. J. Deaf. Stud. Deaf. Educ. 20, 75–81. 10.1093/deafed/enu031, PMID: 25237152

[ref19] Marschark M. (1993). Psychological development of deaf children. (New York: Oxford University Press).

[ref20] Marschark M. Edwards L. Peterson C. Crowe K. Walton D. (2018). Understanding theory of mind in deaf and hearing college students. J. Deaf. Stud. Deaf. Educ. 24, 104–118. 10.1093/deafed/eny039 PMC6436147 30597037

[ref21] Meristo M. Falkman K. W. Hjelmquist E. Tedoldi M. Surian L. Siegal M. (2007). Language and theory of mind reasoning: evidence from deaf children in bilingual and oralist environments. Dev. Psychol. 43, 1156–1169. 10.1037/0012-1649.43.5.1156, PMID: 17723042

[ref22] Mok P. L. H. Pickles A. Durkin K. Conti-Ramsden G. (2014). Longitudinal trajectories of peer relations in children with specific language impairment. J. Child Psychol. Psychiatry 55, 516–527. 10.1111/jcpp.12190, PMID: 24410167 PMC4283728

[ref23] Morgan A. B. Lilienfeld S. O. (2000). A meta-analytic review of the relation between antisocial behavior and neuropsychological measures of executive function. Clin. Psychol. Rev. 20, 113–136. 10.1016/S0272-7358(98)00096-8, PMID: 10660831

[ref24] Perner J. (1991). Learning, development, and conceptual change. Understanding the representational mind. (Cambridge, MA: The MIT Press).

[ref25] Peterson C. C. Siegal M. (2000). Insights into theory of mind from deafness and autism. Mind Lang. 15, 123–145. 10.1111/1468-0017.00126

[ref900] Peterson C. C. Wellman H. M. Liu D. (2005). Steps in theory-of-mind development for children with diversity or autism. Child Dev. 76, 502–517. 15784096 10.1111/j.1467-8624.2005.00859.x

[ref704] Peterson C. C. Wellman H. M. (2009). From infancy to reason: Scaling deaf and hearing children’s understanding of theory of mind and pretence. British Journal of Developmental Psychology 27, 297–310. 19998533 10.1348/026151008x299728 PMC3086259

[ref26] Peterson C. C. Wellman H. M. (2018). Longitudinal theory of mind (ToM) development from preschool to adolescence with and without ToM delay. Child Dev. 10.1111/cdev.13064 29660808

[ref27] Premack D. Woodruff G. (1978). Does the chimpanzee have a theory of mind? Behav. Brain Sci. 1, 515–526. 10.1017/S0140525X00076512

[ref28] Razza R. A. Blair C. (2009). Associations among false-belief understanding, executive function, and social competence: a longitudinal analysis. J. Appl. Dev. Psychol. 30, 332–343. 10.1016/j.appdev.2008.12.020, PMID: 20161159 PMC2735755

[ref29] Russell P. A. Hosie J. A. Gray C. D. Scott C. Hunter N. Banks J. S. (1998). The development of theory of mind in deaf children. J. Child Psychol. Psychiatry 39, 903–910. 10.1111/1469-7610.00390 9758198

[ref705] Schick B. de Villiers P. de Villiers J. Hoffmeister R. (2007). Language and theory of mind: A study of deaf children. Child Development 78, 376–396. 17381779 10.1111/j.1467-8624.2007.01004.x

[ref30] Shakoor S. Jaffee S. R. Bowes L. Ouellet-Morin I. Andreou P. Happé F. (2011). A prospective longitudinal study of children’s theory of mind and adolescent involvement in bullying. J. Child Psychol. Psychiatry 53, 254–261. 10.1111/j.1469-7610.2011.02488.x 22081896 PMC3272094

[ref31] Siegel J. Shaughnessy M. F. (1995). There’s a first time for everything: understanding adolescence. Adolescence 30, 217–221. Available at: http://www.ncbi.nlm.nih.gov/pubmed/7625260. PMID: 7625260

[ref32] Slaughter V. Imuta K. Peterson C. C. Henry J. D. (2015). Meta-analysis of theory of mind and peer popularity in the preschool and early school years. Child Dev. 86, 1159–1174. 10.1111/cdev.12372, PMID: 25874384

[ref706] Spencer P. E. (2010). Play and theory of mind: indicators and engines of early cognitive growth in Oxford handbook of deaf studies, language, and education. eds. Marschark M. Spencer P. E., Vol. 2 (New York: Oxford University Press), 407–424.

[ref2] Suleiman A. B. Harden K. P. (2016). The importance of sexual and romantic development in understanding the developmental neuroscience of adolescence. Dev. Cogn. Neurosci. 17, 145–147. 10.1016/j.dcn.2015.12.007 26778337 PMC6987970

[ref33] St Clair M. C. Pickles A. Durkin K. Conti-Ramsden G. (2011). A longitudinal study of behavioral, emotional and social difficulties in individuals with a history of specific language impairment (SLI). J. Commun. Disord. 44, 186–199. 10.1016/j.jcomdis.2010.09.004, PMID: 20970811

[ref34] Stanzione C. Schick B. (2014). Environmental language factors in theory of mind development. Top. Lang. Disord. 34, 296–312. 10.1097/TLD.0000000000000038

[ref35] Stevenson J. Kreppner J. Pimperton H. Worsfold S. Kennedy C. (2015). Emotional and behavioural difficulties in children and adolescents with hearing impairment: a systematic review and meta-analysis. Eur. Child Adolesc. Psychiatry 24, 477–496. 10.1007/s00787-015-0697-1, PMID: 25758233 PMC4419186

[ref36] Van Gent T. Goedhart A. W. Hindley P. A. Treffers P. D. A. (2007). Prevalence and correlates of psychopathology in a sample of deaf adolescents. J. Child Psychol. Psychiatry 48, 950–958. 10.1111/j.1469-7610.2007.01775.x, PMID: 17714380

[ref37] Vissers C. T. W. M. Hermans D. (2018). Social-emotional problems in deaf and hard-of-hearing children from an executive and theory-of-mind perspective. Oxford Scholarship Online. 10.1093/oso/9780190880545.003.0020

[ref38] Vissers C. Koolen S. (2016). Theory of mind deficits and social emotional functioning in preschoolers with specific language impairment. Front. Psychol. 7:1734. 10.3389/fpsyg.2016.01734, PMID: 27867370 PMC5095688

[ref39] Wadman R. Botting N. Durkin K. Conti-Ramsden G. (2011). Changes in emotional health symptoms in adolescents with specific language impairment. Int. J. Lang. Comm. Disord. 46, 641–656. 10.1111/j.1460-6984.2011.00033.x, PMID: 22026566

[ref40] Wadman R. Durkin K. Conti-Ramsden G. (2008). Self-esteem, shyness, and sociability in adolescents with specific language impairment (SLI). J. Speech Lang. Hear. Res. 51, 938–952. 10.1044/1092-4388(2008/069), PMID: 18658063

[ref41] Yew S. G. K. O’Kearney R. (2013). Emotional and behavioural outcomes later in childhood and adolescence for children with specific language impairments: meta-analyses of controlled prospective studies. J. Child Psychol. Psychiatry Allied Discip. 54, 516–524. 10.1111/jcpp.12009, PMID: 23082773

[ref42] Zaidman-Zait A. Dotan A. (2017). Everyday stressors in deaf and hard of hearing adolescents: the role of coping and pragmatics. J. Deaf. Stud. Deaf. Educ. 22, 257–268. 10.1093/deafed/enw103, PMID: 28334795

